# Ivermectin to reduce malaria transmission: a research agenda for a promising new tool for elimination

**DOI:** 10.1186/1475-2875-12-153

**Published:** 2013-05-07

**Authors:** Carlos J Chaccour, Kevin C Kobylinski, Quique Bassat, Teun Bousema, Chris Drakeley, Pedro Alonso, Brian D Foy

**Affiliations:** 1 Internal Medicine Department, Clínica Universidad de Navarra, Av. Pio XII 36, Pamplona 31008, Spain; 2 Instituto de Salud Tropical, Universidad de Navarra, Pamplona, Spain; 3 Entomology Branch, Walter Reed Army Institute of Research, Silver Spring, Maryland, USA; 4 Barcelona Centre for International Health Research (CRESIB, Hospital Clínic-Universitat de Barcelona), Barcelona, Spain; 5 Centro de Investigação em Saúde de Manhiça (CISM), Maputo, Mozambique; 6 Department of Immunology and Infection, Faculty of Infectious and Tropical Diseases, London School of Hygiene & Tropical Medicine, London, UK; 7 Department of Medical Microbiology, Radboud University, Nijmegen Medical Centre, Nijmegen, The Netherlands; 8 Arthropod-borne and Infectious Diseases Laboratory, Department of Microbiology, Immunology and Pathology, Colorado State University, 1692 Campus Delivery, Fort Collins, Colorado 80523-1692, USA

**Keywords:** Malaria elimination, Ivermectin, Endectocides, Vector control

## Abstract

**Background:**

The heterogeneity of malaria transmission makes widespread elimination a difficult goal to achieve. Most of the current vector control measures insufficiently target outdoor transmission. Also, insecticide resistance threatens to diminish the efficacy of the most prevalent measures, indoor residual spray and insecticide treated nets. Innovative approaches are needed. The use of endectocides, such as ivermectin, could be an important new addition to the toolbox of anti-malarial measures. Ivermectin effectively targets outdoor transmission, has a novel mechanism of action that could circumvent resistance and might be distributed over the channels already in place for the control of onchocerciasis and lymphatic filariasis.

**Methods:**

The previous works involving ivermectin and *Anopheles* vectors are reviewed and summarized. A review of ivermectin’s safety profile is also provided. Finally three definitive clinical trials are described in detail and proposed as the evidence needed for implementation. Several smaller and specific supportive studies are also proposed.

**Conclusions:**

The use of ivermectin solves many challenges identified for future vector control strategies. It is an effective and safe endectocide that was approved for human use more than 25 years ago. Recent studies suggest it might become an effective and complementary strategy in malaria elimination and eradication efforts; however, intensive research will be needed to make this a reality.

## Background

The last 15 years have seen renewed efforts towards controlling malaria-associated morbidity and mortality, eliminating malaria from endemic regions and even planning eventual eradication. For these efforts, several research agendas have been developed [1,2]. However, the gains in malaria control over the last decade are severely threatened by widespread insecticide resistance in vectors [3,4] and emerging artemisinin-resistant *Plasmodium falciparum*[5,6]. These threats pose a major challenge to the vision of the Roll Back Malaria Partnership whereby malaria is no longer a major cause of mortality by 2015 [7]. Global malaria eradication will require new tools and the implementation of an integrated approach targeting the vector and parasite in the ever changing human reservoir [1,8].

Vector control has traditionally been the mainstay of malaria control in the past and is certain to remain so. The Malaria Eradication (malERA) Consultative Group on Vector Control has identified three main challenges to developing vector-targeted interventions that support elimination and eradication goals [9]. The first challenge is developing a broader range of insecticides with novel modes of action to counter current insecticide resistance among *Anopheles* species [10]. Secondly, to develop control methods that affect outdoor feeding and resting vectors; the current most effective tools, indoor residual spraying (IRS) and long-lasting insecticide-treated nets (LLINs), do little to prevent outdoor transmission from these vectors and can even drive exophagy and exophily among them [11]. Lastly, new interventions are needed to reduce the extremely high vectorial capacities of malaria vectors in sub-Saharan Africa [12].

### A potential new tool

Endectocides are drugs that have activity against endoparasites (mainly parasitic nematodes) and ectoparasites, (they can kill arthropods that blood-feed on a treated subject). Ivermectin is the only known endectocide currently approved for human use. It is a semi-synthetic derivate from the fermentation products of *Streptomyces avermectinius*[13]. Ivermectin primarily agonizes glutamate-gated chloride channels in invertebrates, causing flaccid paralysis and death [13]. Glutamate-gated chloride channels do not exist in humans and other weakly sensitive channels are found in the human central nervous system, where the blood–brain barrier limits drug access [14]. These characteristics explain ivermectin’s excellent safety profile (see below). Ivermectin is one of the few drugs used in human mass drug administration (MDA) campaigns, and more than one billion treatments have been delivered over the last 25 years for controlling onchocerciasis and lymphatic filariasis [15].

*Anopheles* mosquitoes are particularly sensitive to very low concentrations of ivermectin relative to other vectors examined [16-18], thus offering promise for malaria control. The methods used to examine ivermectin’s effects on *Anopheles* have been diverse (Table 1), including *in vitro* membrane feeding, direct blood feeding on treated animals or humans [19,20], and wild mosquito collections after they have fed on humans receiving ivermectin MDA [21-23]. These studies clearly show that ivermectin is toxic to all *Anopheles* species examined, and at concentrations found in human blood after treatment.

**Table 1:** **Studies evaluating *Anopheles *mosquito mortality and *Plasmodium *transmission after imbibing blood containing ivermectin**

**Reference**	**Methods**	**Species**	**Results**
Pampiglione 1985 [24]	Feeding on impregnated cotton and on treated mice.	*An. stephensi*	Increased mortality in all groups feeding on impregnated cotton, 100% mortality in those feeding at 28,000 μg/L.
	**Dose:** 140–28,000 μg/kg (once, subcutaneous)		100% mortality 24-hr post feeding on mice treated at dose ≥2,800 μg/kg. Increased mortality in all other dose-groups.
Iakubovich 1989 [25]	Membrane and feeding on treated rabbits. **Dose:** 340 μg/kg (once, subcutaneous)	*An. stephensi*	Death rates among *An. stephensi *fed on rabbits 4, 5 and 6 days after administration of the drug were 93, 70 and 79%, respectively.
		*An. atroparvus*	No difference with control seen in *An. sacharovi *and *An. atroparvus*.
		*An. sacharovi*	
Jones 1992 [26]	Membrane and feeding on treated dogs **Dose: **10–2,500 μg/kg (once, orally)	*An. quadrimaculatus*	Mortality ≥90% in all but one treatment groups 24-hr post blood feeding and ≥90% in all groups 48-hr post blood feeding
		
Gardner 1993 [27]	Feeding on treated dogs	*An. quadrimaculatus*	Significant increase in mortality. LD_50_ = 9.9 μg/kg [6.0, 13.8]
	**Dose:** 6–24 μg/kg (once, orally)		Significant decrease in oviposition and egg-hatching from survivors
Bockarie 1999 [21]	Field collections of engorged females before and after MDA for lymphatic filariasis	*An. punctulatus*	Significant decrease in 9-day cumulative survival rate of *Anopheles* spp. collected 1–3 days post-treatment (0%) *vs *those collected pre-treatment (67%)
	**Dose**: 400 μg/kg ivermectin +/- 6 mg/kg DEC (once, orally)	*An. koliensis*	The 48-hr survival rate of *An. puctulatus *collected from two houses in the a treated village the morning following MDA was 31% *vs *94% from two houses of an untreated village
			Pre- and post-treatment all-night landing catches showed no significant reduction in human biting rates.
Foley 2000 [20]	Feeding on one treated human volunteer **Dose: **250 μg/kg (once, orally)	*An. farauti*	12-day cumulative mortality rate of mosquitoes was 100%, 95%, 93%, and 40% for those fed 0, 7, 10 and 14 days post-treatment *vs *10% for those fed pre-treatment
Fritz 2009 [28]	Membrane and feeding on treated cattle	*An. gambiae*	Membrane feeding: LC_50 _for *An. gambiae *s.l. was 19.8 ± 2.8 ppb; no oviposition from mosquitoes fed on >10 ppb
	**Dose:** 600 μg/kg (once, subcutaneously)	*An. arabiensis*	Cattle feeding: Total cumulative survival of *An. gambiae *s.s. significantly different from controls when fed up to 20 days post-treatment; no or significantly reduced oviposition when fed up to 17 days post-treatment
Chaccour 2010 [19]	Feeding on randomized, treated volunteers and controls		Mean 12-day survival time of 2.38 days [1.52, 3.24] for mosquitoes fed on treated subjects at 1 day post-treatment *vs *5.52 days [4.65, 6.4] for mosquitoes fed on untreated control subjects
	**Dose:** 200 μg/kg (once, orally)	*An. gambiae*	No effect on mosquitoes fed on treated subjects at 14 days post-treatment
Kobylinski 2010 [16]	membrane feedings **Dose:** NA	*An. gambiae*	LC_50_ = 22.4 ng/ml [18.0, 26.9]. At sub-lethal concentrations, significantly reduced mosquito re-blood feeding rates and a second ivermectin blood meal, even at a decreased concentration, further increased mortality
Sylla 2010 [23]	Field collections of engorged females before and after MDA for onchocerciasis	*An. gambiae*	5-day cumulative survival of *An. gambiae s.s. *was significantly reduced from 3 treated villages *vs *pair-matched control villages
	**Dose:** 150 μg/kg (once, orally)	*An. arabiensis*	*An. gambiae s.s. *captured in treated villages 1–6 days post-treatment had significantly reduced survival *v *those caught pre-MDA and those caught >7 days post-treatment
Kobylinski 2011 [22]	Field collections of engorged females before and after MDA for onchocerciasis	*An. gambiae*	For 12 days after the MDA, mean *P. falciparum *sporozoite rate was significantly reduced by 79% in 3 replicate treated villages while it increased by 246% in pair-matched control villages
	**Dose:** 150 μg/kg (once, orally)		
Butters 2012 [29]	Membrane feeding **Dose:** NA	*An. gambiae*	Sub-lethal concentrations (LC_25 _& LC_5_) caused significant knockdown and reduced recovery rates
Fritz 2012 [30]	Membrane feeding **Dose:** NA	*An. arabiensis*	LC_50_ = 7 · 9 ppb [6.2, 9.9]; oviposition among survivors was significantly reduced at ≥7 ppb
Bastiaens 2012 [31]	Feeding on treated Swiss mice, Wistar rats and *Cynomolgus *monkeys **Dose:** 200–400 μg/kg (different intervals, orally)	*An. stephensi*	3-day cumulative mortality of mosquitoes fed on treated mice, rats and monkeys significantly differed from controls when fed up to 2, 4 and 3 days post-treatment, respectively
Kobylinski 2012 [32]	Membrane feeding	*An. gambiae*	Sub-lethal concentrations significantly inhibited *P. falciparum* sporogony when fed prior to, concurrent with, and 6 and 9 days after infection with gametocytes
	**Dose:** NA		

Ivermectin addresses the three main challenges identified by the malERA vector control group [9]: (1) its mode of action is different from the four currently used insecticides for malaria vector control, thus it likely could circumvent the issue of emerging insecticide resistance; (2) as a systemic drug, it is ingested by *all* biting mosquitoes and so it will equally target indoor *and* outdoor-biting mosquitoes, as well as those with crepuscular activity; and, (3) the activity of the drug targets four out of the five variables of vectorial capacity [33,34], especially the most influential variable, the daily probability of mosquito survival. Ivermectin also fits many of the ideals identified in the malERA initiative, including integration with the current vector control tools, any behavioural adaptation away from biting treated humans would only be beneficial, and it is expected to affect vector population structure [35].

### Ivermectin’s safety

#### *The onchocerciasis control programme*

The French authorities approved ivermectin for human use in 1987. Soon after, Merck & Co Inc. decided to donate ivermectin for onchocerciasis control and the Mectizan^®^ Donation Program was created [13]. Since then, more than a 1.5 billion treatments have been distributed in Africa and Latin America for onchocerciasis control and another 665 million for treatment of lymphatic filariasis [15]. In this context, adverse events (AE) to ivermectin have been usually mild, transient, associated with intensity of microfilarial infection and primarily characterized as mild Mazzoti-type reactions to dying microfilaria [36]. No significant association has been found between ivermectin plasma levels and AE [37].

#### *Loa loa*

The limited number of severe neurological AE seen with ivermectin use, include encephalopathy and coma after ivermectin administration to patients who were infected with *Loa loa*. These reactions are closely related to the microfilarial load and are due to parasite lysis rather than drug toxicity [38]. Rapid assessment of loasis is now recommended before ivermectin MDA in *Loa*-endemic areas [39].

#### *Higher or multiple doses*

Several authors have evaluated the safety and tolerability of ivermectin at doses different than those indicated. Duke *et al.*[40] gave adult volunteers six fortnightly doses of 100 μg/kg without observing severe AE. Awadzi *et al.*[41] found no difference with controls in tolerance and early AE using doses of up to 800 μg/kg. Guzzo *et al.*[42] found no significant CNS toxicity or AE in healthy volunteers taking either a single high dose of up to 2,000 μg/kg or repeated doses (three in a week) of up to 1,091 μg/kg. Kamgno *et al.*[43] randomized *Onchocerca*-infected volunteers to different treatment schemes and one group received 800 μg/kg every three months for three years (accumulated dose of 8,950 μg/kg), but reported that all groups had comparable rates of AE. The high dose group reported transitory mild and subjective visual side effects more often (blurring of vision, changes in colour vision, etc.), but ophthalmological examinations revealed no structural explanation.

#### *Children, pregnancy and lactation*

Ivermectin is now licensed for the treatment of children weighing more than 15 kg [44].

Pacque *et al.*[45] carried out a prospective study in Liberia, where 14,000 people received the drug at 150 μg/kg. Out of some 4,000 women treated, 200 were inadvertently treated during pregnancy. No significant differences in birth defect rates, development status or disease patterns could be found when comparing with untreated mothers in the same population. These findings have been confirmed in hundreds of women in North Cameroon [46], Mali [47], Ghana [48] and Uganda [49]. Thus, pregnant women in onchocerciasis-endemic areas at high risk of loss of sight are no longer excluded from ivermectin treatment [50].

Ivermectin levels in human breast milk are low. After a single oral dose of 150 μg/kg in healthy women, Ogbuokiri *et al.*[51] found peak levels of 14.13 +/- 0.43 ng/ml after 6.5 hours. Therefore, a breast-fed new born would get a dose of only 2.75 μg/kg. It is no longer recommended to exclude nursing women during MDA of Mectizan in onchocerciasis-endemic areas [50].

#### *Applying ivermectin for malaria control*

As suggested in the publications in Table 1, and by previous modelling exercises [20,23,35] there are several theoretical ways that ivermectin might be applied to help control malaria:

1) In ongoing onchocerciasis and lymphatic filariasis control campaigns, a single dose of ivermectin is administered to entire villages on a single day during MDA, with coverage rates generally between 60-80%. This has positive collateral effects against soil-transmitted helminthes, [52] and ectoparasites. After MDA, most villagers’ blood is toxic to biting *Anopheles* and this effect may last for approximately six days [23]. These toxic blood meals can kill most of the infectious adult mosquitoes, and while the numbers of adult *Anopheles* feeding on people can rebound quickly depending on the larval reservoir, the new population is young and most have not lived long enough to bite a gametocytaemic person and become infectious (the minimum time required for *P. falciparum* to develop in the vector is nine days). Thus, sporozoite transmission can be suppressed for weeks after MDA [22]. In this way, ivermectin MDA might be ideal to stem malaria epidemics, to interrupt brief transmission seasons or offer sustained transmission reduction if given repeatedly over longer transmission periods.

2) Recent publications have highlighted the likely benefit of combining ivermectin with drugs such as artemisinin combination therapy (ACT). ACT is highly effective in most malaria-endemic settings but does not prevent malaria-transmission in the first weeks after treatment [53,54]. ACT in combination with ivermectin may be an effective option for anti-malarial MDA where residual transmission potential is a major concern [55,56] and where mass screening and treatment (MSAT) or drug combinations fulfilling the single encounter radical cure and prophylaxis (SERCaP) profile [57] are alternative possibilities. Ivermectin would be an additive, blocking onward transmission of parasites from treated individuals by killing most *Anopheles* biting the person and inhibiting *Plasmodium* development in any surviving vectors. This would be especially important to stem the spread of resistant *P. falciparum* and a safe alternative for or in addition to gametocytocidal drugs [58].

3) Treatment of peridomestic animals in areas where *Anopheles* mosquitoes exhibit both zoophagic and anthropophagic behaviour [30], not only with ivermectin but with other classes of systemic endectocides approved for veterinary use, is expected to control the vector population size by increasing mortality, reducing fertility and flying capacity and may have further effects on transmission by inhibiting sporogony in the surviving vectors.

#### *Definitive studies*

Before ivermectin can be recommended for malaria control, large-scale community trials must be conducted to provide definitive evidence of its role in malaria control. Below are what is envisioned as the three primary trials required:

1) Single or repeated human ivermectin MDA for malaria control

Design: placebo-controlled, cluster-randomized, double-blind trial.

Methods: single *vs* repeated ivermectin administration to clusters (villages) over a non-continuous malaria transmission season (e g, the rainy season) at the doses and frequency determined by previous studies.

Entomological measures:

– Mosquito survival

– Mosquito population structure

– Mosquito immigration into clusters

– Entomological inoculation rate

– Vectorial capacity

Parasite measures:

– Sporozoite rates (mosquitoes)

– Plasmodium prevalence through periodic cross-sectionals

– Molecular force of infection

– NTD and ectoparasite prevalence and intensity

Clinical measures:

– Malaria clinical disease incidence as detected by passive case detection

– Serological markers of mosquito bite exposure [59,60]

– Anaemia prevalence

– Adverse events incidence

2) Comparison treatment of ivermectin *vs* ivermectin + ACT

Design: individual-randomized, double-blind trial.

Methods: confirmed cases or asymptomatically infected individuals are enrolled in ACT and ivermectin + ACT arms. Doses and frequency determined by previous studies

Entomological measures:

– Colony mosquito survival and recovery after feeding directly or indirectly on blood of treated subjects

Parasite measures:

– Sporogony assessment in fed mosquitoes

– Plasmodium clearance and rate

– Gametocyte carriage and infectiousness

Clinical measures:

– Safety and toxicology (blood chemistry, haemoglobin, AEs)

– Malaria recovery rate and time to genetically determined new infections after treatments

– PK/PD parameters of ivermectin and ACT

3) Livestock ivermectin/endectocide MDA for malaria control in human population

Design: placebo-controlled, cluster-randomized, double-blind trial.

Methods: repeated ivermectin/endectocide administration to the whole peridomestic livestock population living around clusters. Doses and frequency determined by previous studies

Entomological measures:

–Mosquito survival

– Mosquito population structure/size reduction

– Mosquito immigration into clusters

– Entomological inoculation rate

– Vectorial capacity

Parasite measures:

– Sporozoite rates (mosquitoes)

– Plasmodium prevalence/counts

– Molecular force of infection

– NTD and ectoparasite prevalence/intensity in both animals and humans

Clinical measures:

– Malaria clinical disease incidence as detected by passive case detection

– Anaemia prevalence

#### *Supportive studies*

Numerous studies are needed to fill knowledge gaps about ivermectin’s effects on *Anopheles*, *Plasmodium* and transmission. Some of these studies may be necessary to complete before embarking in more specific clinical trials, such as those proposed above.

### Human plasma levels and mosquito mortality

Current estimates of LC_50_ of ivermectin for mosquitoes are based on membrane feeding essays [16,28,32]. Simultaneous mosquito feeding and measurement of ivermectin concentration in plasma (capillary and venous blood from both men and women) can provide data for a correlation and calculation of *in vivo* LC_50_ and time post-treatment that the anti-mosquito/anti-sporogonic effect lasts. This crucial information, combined with current knowledge of ivermectin’s pharmacokinetics, could be extrapolated into an ideal dosage and spacing of the drug for malaria control. Implied is the standardization of current assays to quantify ivermectin in blood from humans and animals and also the development of sensitive assays to detect and measure the drug in the midgut of fed mosquitoes.

### Confirmation of lethal effects across a range of vector bionomics

The lethal effects of ivermectin on all *Anopheles* species tested so far is expected to extrapolate to exophagic and exophilic vectors, such as *Anopheles minimus* and *Anopheles dirus* in South-East Asia, *Anopheles darlingi* in South America, and newly identified vectors [61]. However, this must be confirmed by well-controlled studies, particularly those assessing effects on wild populations.

### The effects of current ivermectin MDA programmes on malaria transmission

Only a few field trials examining the effects of ivermectin on wild mosquito populations have occurred. They have taken advantage of ongoing once-per-year anti-helminth MDA programmes to assess collateral activity against mosquito survival or changes in the sporozoite rates [22]. Reductions in mosquito survival [21,23] and parasite transmission from single MDA are expected to be temporary, and the degree and duration of these reductions must be thoroughly defined to eventually move to repeat MDA trials (Definitive studies). Furthermore, effects must be compared in diverse habitats containing different vectors and malaria ecologies. In addition to direct anti-mosquito effects, measures need to be made on expected changes in *Anopheles* population structures (as determined by age-grading), and third-order effects on entomological inoculation rate (EIR), vectorial capacity, the molecular force of infection, and the malaria reproductive rate (R_0_).

### Modelling

Very few transmission models have included ivermectin [20,23,35]. Current data suggests that the effect of a single ivermectin dose administered to some 80% of a village’s population could have a profound effect on the age structure of the local *Anopheles* population, reducing transmission for up to three weeks afterwards. Further models fitted from empirical data will be essential to help predict ideal ivermectin MDA dosing and frequency, and the effects MDA might have on local mosquito population dynamics, *Plasmodium* transmission, and human prevalence. Models will be important to predict changes of the above outcomes based on variables such as weather, mosquito immigration, MDA compliance and other concurrent vector control measures.

### Anti-sporogony effects

A recent study [32] has demonstrated that sublethal ivermectin concentrations affect *P. falciparum* transmission by inhibiting sporogony. These data suggest that ivermectin MDA may reduce transmission for a longer period than predicted based on anti-mosquito effects alone, and also enhance ivermectin’s attractiveness to be used in combination with anti-malarials to prevent residual transmission and inhibit the spread of anti-malarial resistance. This study needs confirmation using wild parasite isolates and with other *Anopheles* and *Plasmodium* species.

### Safety and formulation assessments

A single ivermectin MDA with coverage around 80% disrupts malaria transmission in a community by changing the structure of the local mosquito population [22,23]. The effect is longer than expected given the current drug formulation´s short half-life. Modelling [23,35] suggests that intermittent repeated administration would sustain control while minimizing mosquito and helminth resistance development. However, an alternative, single-encounter, long-lasting formulation could be less costly and more logistically feasible in MDA or MSAT approaches, and likely superior in individualized treatments meant to contain the spread of artemisinin-resistant *Plasmodium*. While the current ivermectin formulation should be tested over the short term, longer-term research should focus on finding a formulation capable of safely maintaining zero-order release for a period long enough to have a lasting impact on the malaria reproductive number (R_0_). Concurrent toxicity studies must be done and possible interactions with anti-malarials and other commonly used drugs assessed.

### Emerging resistance

Ivermectin is of capital importance for the control of onchocerciasis, lymphatic filariasis and for the treatment of some soil-transmitted helminths. Increasing the exposure of parasites to the drug is likely to lead to resistance in other parasites and jeopardize the success of control programmes. Indeed, ivermectin resistance has been documented in *Sarcoptes scabiei* in Australia and possibly in *Onchocerca volvulus* in Ghana [62]. Ongoing surveillance must be established in communities where ivermectin is introduced as a malaria control measure and plausible methods to delay or reverse resistance explored. Combination therapy with a second anthelmintic, such as a benzmidazole, might be effective. Likewise, proper dosing and MDA spacing might delay ivermectin resistance development in soil-transmitted helminths.

Ivermectin has a mechanism of action unrelated to that of commonly used insecticides in malaria-endemic regions. However, there must be early research on the possibility of resistance mechanisms in *Anopheles*, a better understanding of the molecular targets of ivermectin in the mosquito and possible metabolic detoxification mechanisms that could foster cross-resistance.

#### Final comments

Available vector control tools are not effective enough to achieve widespread malaria elimination or eradication, and innovative approaches are needed. The use of ivermectin solves many challenges identified for future vector control strategies. It is an effective and safe endectocide that was approved for human use more than 30 years ago. Recent studies suggest it might become an effective and complementary strategy in malaria elimination and eradication efforts; however, intensive research will be needed to make this a reality.

## Competing interests

The authors declare that they have no competing interests.

## Authors’ contributions

CJC and BDF wrote the first draft. All authors contributed and approved the manuscript.
